# Primary Cytoreduction and Survival for Patients With Less-Common Epithelial Ovarian Cancer

**DOI:** 10.1001/jamanetworkopen.2024.17775

**Published:** 2024-06-20

**Authors:** Koji Matsuo, Ling Chen, Maximilian Klar, Lynda D. Roman, Anil K. Sood, David M. Gershenson, Jason D. Wright

**Affiliations:** 1 Division of Gynecologic Oncology, Department of Obstetrics and Gynecology, University of Southern California, Los Angeles; 2 Norris Comprehensive Cancer Center, University of Southern California, Los Angeles; 3 Division of Gynecologic Oncology, Department of Obstetrics and Gynecology, Columbia University College of Physicians and Surgeons, New York, New York; 4 Department of Obstetrics and Gynecology, University Medical Center Freiburg, University of Freiburg, Freiburg, Germany; 5 Department of Gynecologic Oncology and Reproductive Medicine, The University of Texas MD Anderson Cancer Center, Houston

## Abstract

This cohort study examines the association between primary cytoreduction status and survival for patients with less-common, advanced-stage epithelial ovarian carcinoma.

## Introduction

For advanced stage ovarian cancer, surgery to perform maximal tumor cytoreduction has been the mainstay of initial therapy.^1^ The extent of residual disease at the completion of surgery is highly prognostic, with lower-volume residual disease associated with improved survival.^2^

Prior studies examining the association between cytoreductive effort and survival have included mainly high-grade and serous ovarian tumors, the most common histologic subtype of ovarian cancer.^2^ The prognostic significance of cytoreductive status in less-common histologic types has been relatively understudied.

Given the distinct clinical and biological differences across histologic subtypes in epithelial ovarian cancer,^3,4^ data derived mainly from common high-grade serous tumors may not apply to less-common histologic types. The objective of this study was to examine the association between primary cytoreduction status and survival for patients with less-common, advanced-stage epithelial ovarian carcinoma.

## Methods

This cohort study was deemed exempt by the Columbia University institutional review board with a waiver of informed consent because it included only publicly available, deidentified data. This study is reported following the Strengthening the Reporting of Observational Studies in Epidemiology (STROBE) reporting guideline. The study population included patients with stage III epithelial ovarian carcinomas who underwent primary cytoreductive surgery from 2011 to 2020, with data collected from the National Cancer Database.^5^ The association of residual disease at the completion of primary cytoreduction (complete cytoreduction [R0], optimal cytoreduction [R1], and suboptimal cytoreduction [R2]) and overall survival (OS) were assessed with Cox proportional hazards regression models, adjusting for other clinical and demographic characteristics. *P* values were 2-tailed, and statistical significance was set at *P* < .05. Analyses were conducted using SAS software version 9.4 (SAS Institute). Further details are provided in the eMethods in Supplement 1. Analyses were conducted from October 2023 to February 2024.

## Results

A total of 1100 patients with ovarian clear cell carcinoma (CCC), 495 patients with mucinous ovarian carcinoma (MOC), 1103 patients with low-grade serous ovarian carcinoma (LGSOC), and 13 046 patients with high-grade serous ovarian carcinoma (HGSOC) were examined for analysis. Complete cytoreduction rates were overall higher in less-common cancer types compared with HGSOC (73.2% for LGSOC, 74.0% for CCC, 72.1% for MOC, and 59.9% for HGSOC; *P* < .001). Complete cytoreduction rates increased during the study period in HGSOC (52.2% to 62.8%; relative increase, 27.8%; *P* < .001), but not in less-common cancers (CCC, 60.5% to 77.3%; *P* = .32; MOC, 60.0% to 70.7%; *P* = .18; LGSOC, 67.0% to 70.1%; *P* = .48) (Figure, A). Across the 4 histologic types, the annual number of primary cytoreductive surgical procedures decreased by 41.6% from 2011 to 2020 (1811 to 1057 procedures) (Figure, A).

**Figure.**
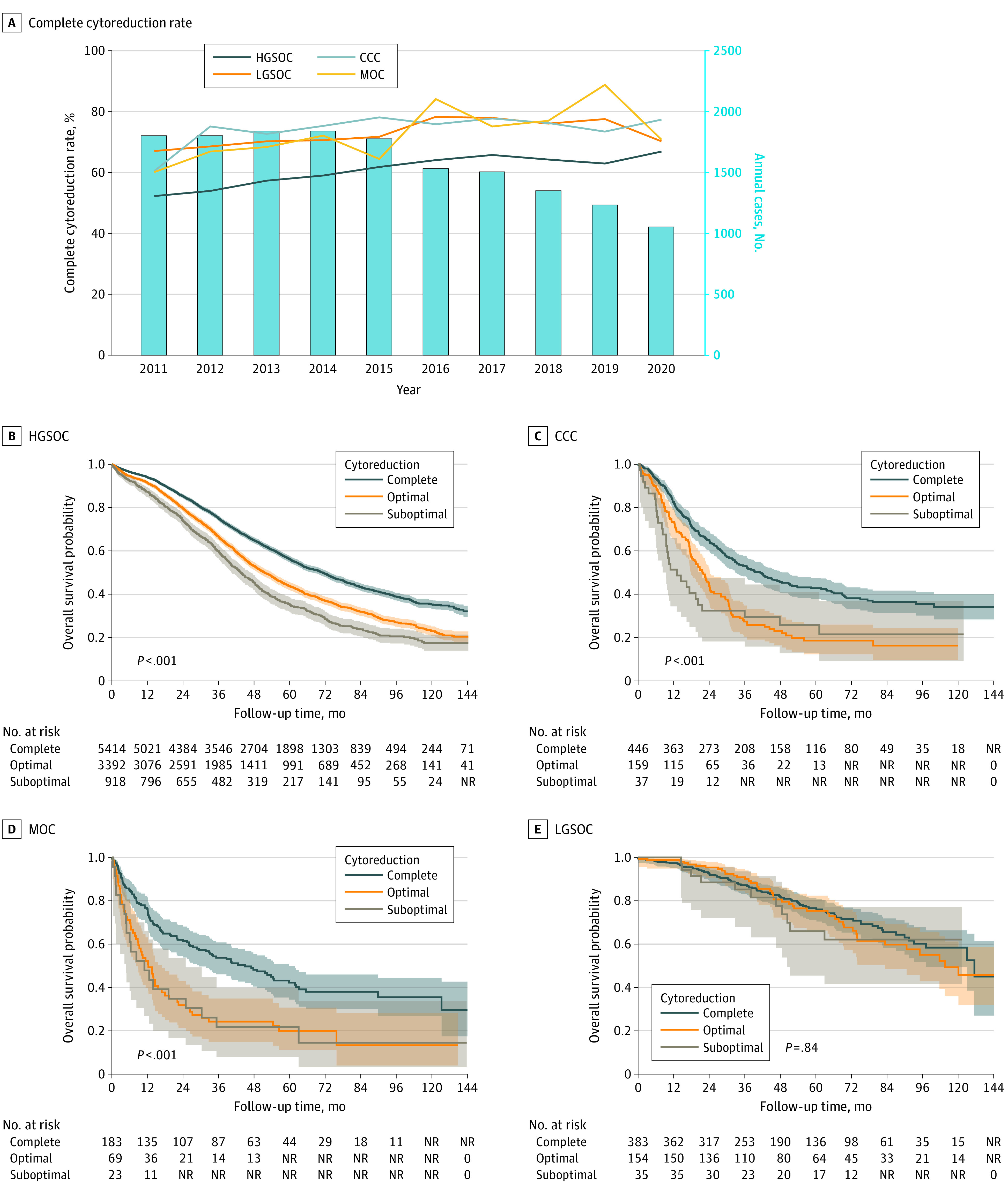
Temporal Trends and Survival Outcomes Related to Cytoreduction Status A, Lines represent complete cytoreduction rates at primary cytoreductive surgery for stage III disease and are shown for 4 histologic types from 2011 to 2020. Bars represent annual cases of primary cytoreductive surgery for these histological types. B-E, Assessment of stage IIIC disease based on cytoreduction status. Survival data were available for 2011 to 2019. CCC indicates clear cell carcinoma; HGSOC, high-grade serous ovarian carcinoma; LGSOC, low-grade serous ovarian carcinoma; and MOC, mucinous ovarian carcinoma; NR, not reported (data suppressed owing to small cell count).

In the HGSOC cohort, larger residual disease after primary cytoreduction was associated with decreased OS (5-year rates: R0, 58.5%; R1, 45.0%; R2, 36.2%; R1 vs R0 adjusted hazard ratio [aHR], 1.44; 95% CI, 1.36-1.52; R2 vs R0 aHR, 1.77; 95% CI, 1.63-1.93) (Table). Results remained consistent in stage IIIC disease (Figure, B).

**Table.** Survival Estimates per Cytoreduction Status

Cytoreduction	HGSOC		CCC		MOC		LGSOC	
	5-y OS, % (95% CI)	aHR (95% CI)	5-y OS, % (95% CI)	aHR (95% CI)	5-y OS, % (95% CI)	aHR (95% CI)	5-y OS, % (95% CI)	aHR (95% CI)
**Overall** ^a^								
Complete	58.5 (57.2-59.7)	1 [Reference]	45.2 (41.1-49.1)	1 [Reference]	40.5 (34.6-46.2)	1 [Reference]	80.8 (76.8-84.2)	1 [Reference]
Optimal	45.0 (43.3-46.7)	1.44 (1.36-1.52)^b^	25.9 (19.7-32.6)	1.72 (1.42-2.08)^b^	25.1 (16.7-34.4)	1.55 (1.17-2.07)^b^	75.3 (67.4-81.6)	1.04 (0.75-1.45)
Suboptimal	36.2 (33.0-39.5)	1.77 (1.63-1.93)^b^	29.5 (16.6-43.7)	1.95 (1.35-2.81)^b^	26.3 (11.8-43.4)	1.63 (1.03-2.56)^b^	63.0 (43.6-77.3)	1.19 (0.66-2.12)
**Stage IIIC disease** ^c^								
Complete	56.2 (54.8-57.7)	1 [Reference]	42.8 (37.9-47.6)	1 [Reference]	42.2 (34.4-49.9)	1 [Reference]	76.6 (71.1-81.2)	1 [Reference]
Optimal	43.8 (42.0-45.6)	1.42 (1.34-1.50)^b^	18.6 (12.3-26.0)	1.82 (1.46-2.27)^b^	20.0 (11.1-30.7)	1.86 (1.33-2.59)^b^	75.4 (66.2-82.3)	1.08 (0.75-1.54)
Suboptimal	35.4 (32.0-38.8)	1.74 (1.59-1.90)^b^	25.8 (12.8-41.0)	2.04 (1.38-3.02)^b^	21.7 (7.9-39.9)	1.89 (1.15-3.11)^b^	66.0 (45.5-80.3)	1.06 (0.56-1.98)

Abbreviations: aHR, adjusted hazard ratio; CCC, clear cell carcinoma; HGSOC, high-grade serous ovarian carcinoma; LGSOC, low-grade serous ovarian carcinoma; MOC, mucinous ovarian carcinoma; OS, overall survival.

^a^ Calculated using Cox proportional hazard regression model: exposure-outcome association was adjusted for age, race and ethnicity, comorbidity index, cancer stage, debulking procedure with intestinal or urinary tract resection, and postoperative chemotherapy.

^b^ *P* < .05.

^c^ Adjusted for age in these subcohorts.

In the CCC and MOC cohorts, complete cytoreduction was associated with improved OS (5-year rates for CCC: R0, 45.2%; R1, 25.9% R2, 29.5%; R1 vs R0 aHR, 1.72; 95% CI, 1.42-2.08; R2 vs R0 aHR, 1.95; 95% CI, 1.35-2.81). Five-year OS rates for MOC were 40.5% for R0, 25.1% for R1 (R1 vs R0 aHR, 1.55; 95% CI, 1.17-2.07), and 26.3% for R2 (R2 vs R0 aHR, 1.63; 95% CI, 1.03-2.56) (Table). These survival associations remained in stage IIIC disease (Figure, C and D; Table).

For the LGSOC cohort, the association between the extent of cytoreduction and OS was not statistically significant. Five-year OS rates were 80.8% for R0, 75.3% for R1 (R1 vs R0 aHR, 1.04; 95% CI, 0.75-1.45), and 63.0% for R2 (R2 vs R0 aHR, 1.19; 95% CI, 0.66-2.12) (Table). The finding was similar in stage IIIC disease (Figure, E), with 5-year OS rates of 76.6% for R0, 75.4% for R1 (R1 vs R0 aHR, 1.08; 95% CI, 0.75-1.54), and 66.0% for R2 (R2 vs R0 aHR, 1.06; 95% CI, 0.56-1.98) (Table).

## Discussion

This cohort study found that the association of residual tumor volume at primary cytoreduction with survival was less robust in LGSOC compared with other histology types. The decreasing use of primary cytoreductive surgery likely reflects an increasing use of neoadjuvant chemotherapy for advanced-stage ovarian cancer. The increase in use of neoadjuvant chemotherapy has also been demonstrated for LGSOC.^6^ However, neoadjuvant chemotherapy is associated with decreased survival for advanced-stage LGSOC compared with primary cytoreduction.^6^

While effort should be made to remove as much tumor as is feasible for patients with LGSOC, the findings of this cohort study suggest that the survival benefit of achieving R0 status is less robust for LGSOC than for HGSOC, CCC, or MOC. Primary cytoreduction is thus preferred for advanced LGSOC, even complete or optimal cytoreduction is challenging, and use of neoadjuvant chemotherapy would need to be limited as possible.

Key limitations in this study included unmeasured confounding with lack of information on tumor burden and complexity, anatomical location, and exact size of residual disease. Also, recurrence data were not available in the database. Despite these limitations, the observed survival associations call for awareness of the distinct clinical and biological differences across epithelial ovarian carcinoma histological types, particularly for LGSOC.^3,4^

## Notes

Supplement 1.
eMethods.

Supplement 2.
Data Sharing Statement
