# Supplementary material for: Primary Cytoreduction and Survival for Patients With Less-Common Epithelial Ovarian Cancer
Source: JAMA Netw Open. 2024 Jun 20;7(6):e2417775. doi: 10.1001/jamanetworkopen.2024.17775 (PMC11190790; doi:10.1001/jamanetworkopen.2024.17775)
Supplement: Supplement 1. — eMethods. [file jamanetwopen-e2417775-s001.pdf]

Supplemental Online Content

Matsuo K, Chen L, Klar M, et al. Primary Cytoreduction and Survival for Patients With Less-Common Epithelial Ovarian Cancer. JAMA Netw Open. 2024;7(6):e2417775. doi:10.1001/jamanetworkopen.2024.17775

eMethods.

This supplemental material has been provided by the authors to give readers additional information about their work.

© 2024 Matsuo K et al. *JAMA Network Open.*

eMethods. Materials and Methods.

This retrospective cohort study utilized the Commission-on-Cancer's National Cancer Database.[1] The Columbia University Institutional Review Boards deemed the study non-human subjects research as it included only publicly available, deidentified data.

The study population included patients with stage III epithelial ovarian carcinomas who underwent primary cytoreductive surgery from 2011-2020. Less-common tumor subtypes based the National Comprehensive Cancer Network definition[2] included clear cell carcinoma (CCC), mucinous ovarian carcinoma (MOC), and low-grade serous ovarian carcinoma (LGSOC). Patients with high-grade serous ovarian carcinoma (HGSOC) served as the control group. Patients with stage IV tumors and those who received neoadjuvant chemotherapy were not included.

Histologic subtypes were based on the World Health Organization's International Classification of Diseases for Oncology, third edition: CCC (8005/3, 8310/3, 8313/3, 8443/3, and 8444/3), MOC (8470/3, 8471/3, 8472/3, 8480/3, 8481/3, 8482/3, and 9015/3), LGSOC (8441/3, 8442/3, 8460/3, 8461/3, 8462/3, and 9014/3) with well-differentiated lesions, and HGSOC (8441/3, 8442/3, 8460/3, 8461/3, 8462/3, and 9014/3) with moderately and poorly deafferentations. These grouping followed prior investigation.[3]

The exposure was the extent of residual disease at the completion of primary cytoreduction, classified as complete cytoreduction (no gross residual disease [R0]), optimal cytoreduction (no more than 1 cm residual disease [R1]), and suboptimal cytoreduction (greater than 1 cm residual disease [R2]).[2] These were allocated based on CS Site-Specific Factor 3 Residual Tumor Status and Size After Primary Cytoreduction Surgery (CS_SITESPECIFIC_FACTOR_3) for 2004-2017 and Residual Tumor Volume Post Cytoreduction (RESID_POST_CYTOREDU) for 2018-2020.[4,5]

The main outcome was overall survival (OS). Kaplan-Meier curves were developed for each histologic sub-type and differences in survival based on cytoreductive status was compared using log-rank tests. Cox proportional hazards regression models were developed to estimate the association between cytoreductive status and OS while adjusting for other clinical and demographic characteristics. Sensitivity analyses included evaluation of stage IIIC disease as this group is the most frequent sub-stage for advanced ovarian cancer.

© 2024 Matsuo K et al. *JAMA Network Open.*

Statistical interpretation was based on a two-tailed hypothesis, and a $P<.05$ was considered statistically significant. SAS Software version 9.4 (SAS Institute Inc., Cary, NC, USA) was used for the statistical analysis. The STROBE reporting guidelines were followed to summarize the performance of cohort study.[6] Statistical analysis was performed from 10/2023-2/2024.

EFERENECES

1. National Cancer Database. American College of Surgeons. <https://www.facs.org/quality-programs/cancer-programs/national-cancer-database/> (accessed 2/16/2024).

2. Ovarian cancer including fallopian tube cancer and primary peritoneal cancer. NCCN Clinical Practice Guidelines in Oncology (NCCN Guidelines). National Comprehensive Cancer Network. Version 1.2024. <http://www.nccn.org/guidelines>. (accessed 2/16/2024).

3. Matsuo K, Matsuzaki S, Maeda M, Rau AR, Yoshihara K, Tamura R, Shimada M, Machida H, Mikami M, Klar M, Roman LD, Wright JD, Sood AK, Gershenson DM. Uptake and Outcomes of Neoadjuvant Chemotherapy Among US Patients With Less Common Epithelial Ovarian Carcinomas JAMA Netw Open. 2023;6:e2318602. doi: 10.1001/jamanetworkopen.2023.18602.

4. https://web2.facs.org/cstage0205/ovary/Ovary_lpt.html

5.https://staging.seer.cancer.gov/eod_public/input/2.1/fallopian_tube/resid_tumor_vol_post_cyto/?breadcrumbs=(~view_schema~,~fallopian_tube~)

6. http://www.equator-network.org/reporting-guidelines/strobe/

© 2024 Matsuo K et al. *JAMA Network Open.*
