# Supplementary material for: Primary Cytoreduction and Survival for Patients With Less-Common Epithelial Ovarian Cancer
Source: JAMA Netw Open. 2024 Jun 20;7(6):e2417775. doi: 10.1001/jamanetworkopen.2024.17775 (PMC11190790; doi:10.1001/jamanetworkopen.2024.17775)
Supplement: Supplement 2. — Data Sharing Statement [file jamanetwopen-e2417775-s002.pdf]

Data Sharing Statement

Matsuo. Primary Cytoreduction and Survival for Patients With Less-Common Epithelial Ovarian Cancer. *JAMA Netw Open*. Published June 20, 2024.
doi:10.1001/jamanetworkopen.2024.17775

Data
Data available: No

Additional Information
Explanation for why data not available: Data availability statement: The data on which this study is based are American College of Surgeons National Cancer Database (https://www.facs.org/quality-programs/cancer-programs/national-cancer-database/).
